# Hepatic Metabolic Profile Reveals the Adaptive Mechanisms of Ewes to Severe Undernutrition during Late Gestation

**DOI:** 10.3390/metabo8040085

**Published:** 2018-11-27

**Authors:** Yanfeng Xue, Changzheng Guo, Fan Hu, Junhua Liu, Shengyong Mao

**Affiliations:** Jiangsu Key Laboratory of Gastrointestinal Nutrition and Animal Health, Laboratory of Gastrointestinal Microbiology, College of Animal Science and Technology, Nanjing Agricultural University, Nanjing 210095, China; xueyanfeng1990@163.com (Y.X.); 2017205025@njau.edu.cn (C.G.); 2016105046@njau.edu.cn (F.H.); liujunhua0011@163.com (J.L.)

**Keywords:** undernutrition, adaptive mechanisms, lipid metabolism, fatty acid, amino acid

## Abstract

The mechanisms underlying the adaption of liver metabolism to the undernutrition in ewes during late gestation remain unclear. This research aimed to explore the adaptive mechanisms of liver metabolism by hepatic metabolome analysis in pregnant ewes to the negative energy balance induced by severe feed restriction. Twenty ewes carrying multiple fetuses and gestating for 115 days were fed normally or restricted to a 30% feed level (10 ewes in each group) for 15 days. All ewes were sacrificed and hepatic samples were collected and analyzed by liquid chromatography-mass spectrometry. Both the principal components analysis and partial least squares of discriminant analysis of hepatic metabolites showed the clear separation between ewes in the control and severely feed-restricted groups. The metabolic profile demonstrated that the proportions of differential metabolites between the two groups in fatty acids and lipids, organic acids, and amino acids and derivatives were 61.11%, 16.67%, and 11.11%, respectively. Enriched pathways of differential metabolites were mainly involved in fatty acids and amino acids metabolism and biosynthesis. Correlation networks of differential metabolites revealed that general metabolic pattern was changed apparently and mainly based on fatty acids and lipids in the livers of feed-restricted ewes. The accumulation and oxidation of long-chain fatty acids were intensified in the livers of feed-restricted ewes, while those of medium-chain fatty acids were the opposite. In general, severe feed restriction significantly affected the levels of hepatic metabolites and altered the overall metabolic pattern. Furthermore, fatty acids oxidation as well as the utilization of amino acids and organic acids were intensified to adapt to the negative energy balance during late gestation.

## 1. Introduction

Compared with maintenance level, energy requirements of ewes in late stages of pregnancy are increased by 150% or 200% for sheep carrying a single fetus or twins, respectively [1]. The reason is that approximately 80% of the fetal growth proceeds during this period. However, in addition to the apparently elevated energy requirement, dry matter intake is decreased in advanced pregnancy, mainly due to the reduction of ruminal capacity resulting from the increased volume of the uterus [2]. Particularly, ewes carrying two or more fetuses are more susceptible to negative energy balance because of the much higher energy demands and much lower feed intakes.

To provide nutrients for fetal growth and development, the maternal body is able to adaptively adjust the physiological metabolism of fats, carbohydrates, and proteins through the endocrine system [3,4]. However, the alternations of metabolic status may disrupt maternal metabolic homeostasis. With a continuously decreased nutrition supply, this protective mechanism results in the hypoglycemia and the extensive production of ketone bodies in the maternal body [5]. When this proceeds too rapidly, the body can’t clear ketone bodies in a timely manner, and pregnancy toxemia occurs [6,7]. Pregnancy toxemia is always associated with a high mortality rate for the mother and fetus, resulting in huge economic losses in ewe livestock production [6,8]. Despite the broad research on feed restriction and pregnancy toxemia for ewes, their adaptive mechanisms to severe undernutrition during late pregnancy are poorly understood. In the recent years, the development and marketization of metabolome technology has obviously facilitated the sensitively unbiased evaluation of global metabolites, which could be crucial to understanding these complex adaptive mechanisms.

Here, a hypothesis is proposed that the metabolism of lipids, amino acids, and organic acids as well as even the general metabolic pattern will be altered in severely feed-restricted ewes during advanced pregnancy to adapt to the undernutrition. Thus, the current study aimed to characterize the hepatic metabolic changes in ewes with severe feed restriction through a metabolome analysis to further explore these adaptive mechanisms.

## 2. Results

### 2.1. Performance and Indicators

**Figure 1:**
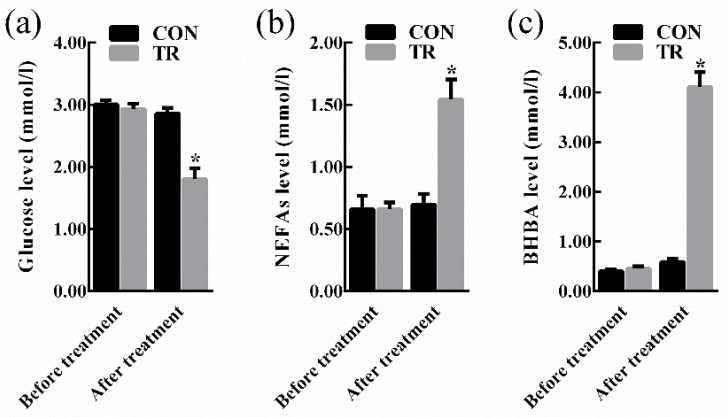
Blood biochemical indicators of ewes in the control group (CON, fed at the normal level, *n* = 10) and treated group (TR, restricted to a 30% level of feed intake, *n* = 10) before and after intervention. (**a**) Glucose level. (**b**) NEFAs level. (**c**) BHBA level. Values are means with their standard errors represented by vertical bars. * Mean values with asterisks were significantly different between the two groups (*p* < 0.05, independent-sample *t*-test). NEFAs, non-esterified fatty acids; BHBA, beta-hydroxybutyric acid.

**Figure 2:**
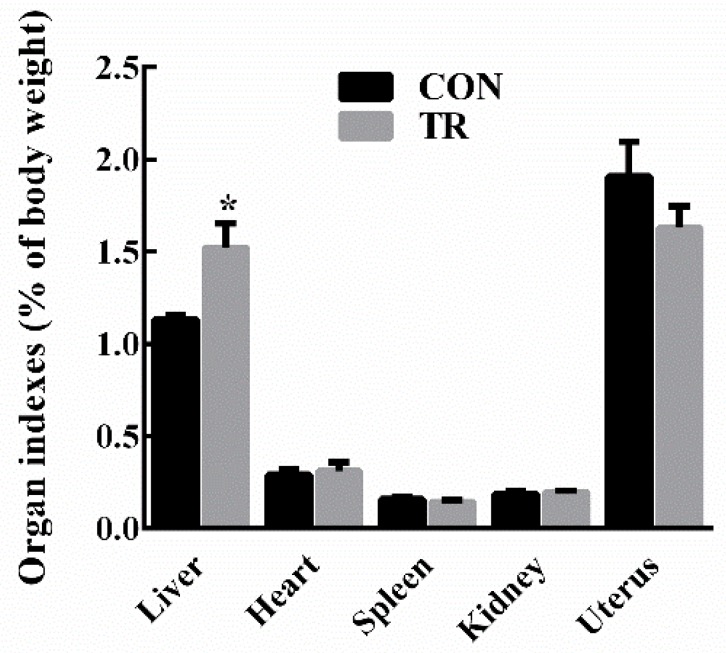
Organ indexes of ewes in the control group (CON, fed at the normal level, *n* = 10) and treated group (TR, restricted to a 30% level of feed intake, *n* = 10). Values are means with their standard errors represented by vertical bars. * Mean values with asterisks were significantly different between the two groups (*p* < 0.05, independent-sample *t*-test).

At the end of experiment, all ewes in the treated (TR) group displayed clinical symptoms of pregnancy toxemia including apathy, anorexia, weakness, empty chewing movements, grinding teeth, dyskinesia, while ewes in the control (CON) group kept alive and alert. As shown in Figure 1, there were no significant differences in the levels of blood glucose, non-esterified fatty acids (NEFAs), and beta-hydroxybutyric acid (BHBA) between the two groups before intervention. After intervention, in the TR group, the blood glucose level was lower (*p* < 0.05), while the blood NEFAs level and BHBA level were higher (*p* < 0.05) than those of the CON group, respectively. Results of maternal organ indexes showed that liver index of the TR group was greater (*p* < 0.05) than that of the CON group. While heart index, spleen index, kidney index, and uterus index of the TR group remained unchanged (*p* > 0.05) when compared with those of the CON group (Figure 2). As shown in Supplementary Figure S1a, the livers of ewes in the CON group were dark red, while the livers of ewes in the TR group were deep yellow and extremely swollen. To explore the reason for the alteration of hepatic appearance, hematoxylin-eosin stained histologic sections were made. Results showed that severe feed restriction extremely increased fat vacuoles in hepatic sections and the nuclei were surrounded by fat vacuoles (Supplementary Figure S1c) when compared with the CON group (Supplementary Figure S1b).

### 2.2. Identification and Quantification of Compounds

In hepatic tissue samples, 301 unique and non-overlapping valid peaks were detected by liquid chromatography-mass spectrometry (LC/MS). Following the rigorous quality checks and identification, 155 metabolites across all samples were obtained, which were primarily fatty acids, lipids, sugars, amino acids, organic acids, nucleotides, and vitamins.

**Figure 3:**
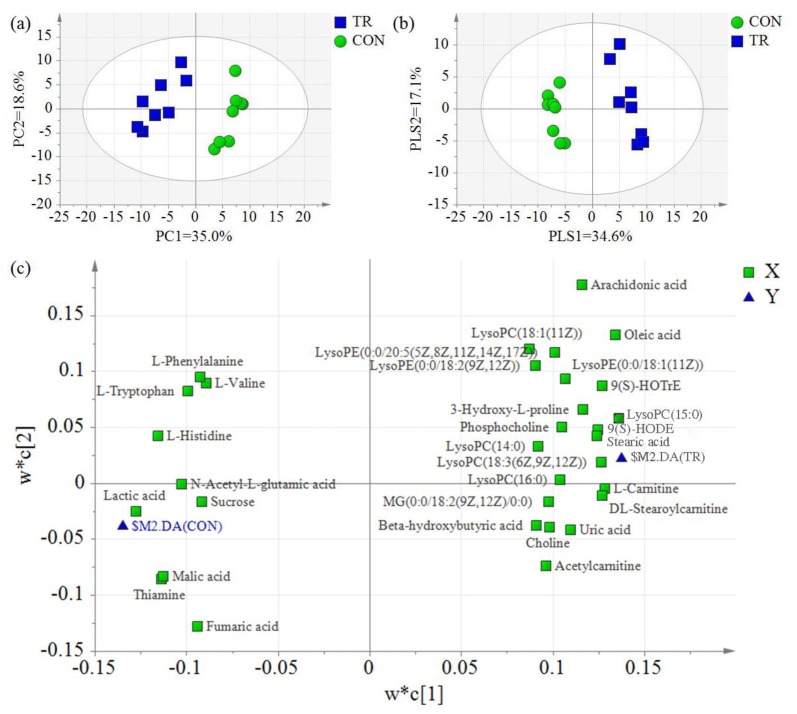
Principal components analysis (PCA) and partial least squares of discriminant analysis (PLS-DA) of hepatic metabolites for ewes in the control group (CON, fed at the normal level, *n* = 8) and treated group (TR, restricted to a 30% level of feed intake, *n* = 8). (**a**) PCA score scatter plot; (**b**) PLS-DA score scatter plot (predictive ability parameter (Q^2^) (cum) = 0.945, goodness-of-fit parameter (R^2^) (Y) = 0.980); (**c**) PLS-DA loading scatter plot. Only the differential metabolites distributed near to the $M2.DA(CON) or $M2.DA(TR) were shown in the loading scatter plot.

For further analysis, the principal component analysis (PCA) and partial least squares discriminant analysis (PLS-DA) of hepatic metabolites in the CON and TR groups were conducted. As expected, the hepatic metabolites of feed-restricted ewes were clearly separated from those of the CON group according to the PCA. The principal coordinate analysis displayed that PCA axis 1 and axis 2, respectively, explained 35.0% and 18.6% of the total variation (Figure 3a). Similar to the PCA results, two groups were also separated clearly by the PLS-DA, and the PLS-DA axis 1 and axis 2 explained 34.6% and 17.1% of the total variation, respectively (Figure 3b). The PLS-DA loading scatter plot demonstrated the distribution of hepatic metabolites. By integrating the PLS-DA score scatter plot and loading scatter plot, it was found that some compounds, including lysophosphatidyl choline (LysoPC) (14:0), LysoPC(15:0), LysoPC(16:0), LysoPC(18:1(11*Z*)), lysophosphatidyl ethanolamine (LysoPE)(0:0/18:1(11*Z*)), LysoPE(0:0/18:2(9*Z*,12*Z*)), LysoPE(0:0/20:5(5*Z*,8*Z*,11*Z*,14*Z*,17*Z*)), l-carnitine, dl-stearoylcarnitine, acetylcarnitine, 9*S*-hydroxy-10*E*,12*Z*-octadecadienoic acid (9(*S*)-HODE), 9*S*-hydroxy-10*E*,12*Z*,15*Z*-octadecatrienoic acid (9(*S*)-HOTrE), stearic acid, and oleic acid, were positively related with feed-restricted ewes, while other compounds, including l-histidine, l-valine, l-tryptophan, l-phenylalanine, *N*-acetyl-l-glutamic acid, lactic acid, malic acid, fumaric acid, sucrose, and thiamine, were positively related with ewes in the CON group (Figure 3c).

### 2.3. Differential Hepatic Metabolites

Combined with false discovery rate (FDR), variable importance in projection (VIP), and fold change (FC), 54 differential metabolites were identified in total (FDR < 0.05, VIP > 1, and FC > 1.5 or < 0.67). Subsequently, differential hepatic metabolites were classified according to their properties. Details of the differential metabolites, including name, VIP, FDR, and FC, are represented in Table 1. Generally, the results showed that the main differences between hepatic metabolites of feed-restricted ewes and ewes in the CON group were the alterations of fatty acids and lipids (61.11%), amino acids and derivatives (11.11%), sugars (4.84%), organic acids (16.67%), and nucleosides and nucleotides (6.45%). Compared with ewes in the CON group, feed restriction increased the levels of 28 hepatic metabolites and decreased the levels of 26 hepatic metabolites.

As shown in Table 1, a significant increase in the levels of long-chain fatty acids, such as 9(*S*)-HODE, 9(*S*)-HOTrE, oleic acid, linoleic acid, stearic acid, and arachidonic acid, was observed, while a significant decrease in the levels of medium-chain fatty acids, including dl-2-aminooctanoic acid, cinnamic acid, and 2-hydroxycinnamic acid, was observed in the hepatic tissues of feed-restricted ewes, in comparison with ewes in the CON group. As the end-product of acetyl-CoA incomplete oxidation, BHBA in the hepatic tissues of feed-restricted ewes was markedly higher than that of the CON group. Meanwhile, an extremely apparent alteration of lipids levels was found in the present study. Compared with ewes in the CON group, six species of LysoPC, three species of LysoPE, one specie of monoacylglycerol (MG), and two species of choline, including LysoPC(14:0), LysoPC(15:0), LysoPC(16:0), LysoPC(18:1(11*Z*)), LysoPC(18:3(6*Z*,9*Z*,12*Z*)), LysoPC(20:4(5*Z*,8*Z*,11*Z*,15*Z*)), LysoPE(0:0/18:1(11*Z*)), LysoPE(0:0/18:2(9*Z*,12*Z*)), LysoPE(0:0/20:5(5*Z*,8*Z*,11*Z*,14*Z*,17*Z*)), MG(0:0/18:2(9*Z*,12*Z*)/0:0), acetylcholine, and phosphocholine, were significantly increased, while only one specie of LysoPCs and two species of LysoPE, including LysoPC(22:4(7*Z*,10*Z*,13*Z*,16*Z*)), LysoPE(0:0/15:0), and LysoPE(0:0/18:3(9*Z*,12*Z*,15*Z*)), were significantly decreased in the hepatic tissues of feed-restricted ewes. The levels of acetyl-l-carnitine, l-carnitine, and stearoylcarnitine in the hepatic tissues of feed-restricted ewes were 135.3-, 7.2-, and 14.8-folds greater, respectively, than those of ewes in the CON group.

Regarding to the altered amino acids and derivatives, five of them, including l-histidine, l-valine, l-tryptophan, l-phenylalanine, and *N*-acetyl-l-glutamic acid, were decreased, while the level of 3-hydroxy-l-proline was enriched in the hepatic tissues of feed-restricted ewes when compared to ewes in the CON group. For sugars, the results revealed that feed-restricted ewes had a significantly lower level of sucrose compared with ewes in the CON group. Among the nucleosides and nucleotides, as compared with ewes in CON group, feed restriction caused a decrease in the levels of hypoxanthine, 5-methylcytidine, and inosine in the liver. In addition, thiamine was the only vitamin identified as a differential metabolite, which was just 25.5% in feed-restricted ewes, relative to that of ewes in the CON group. With respect to organic acids, the levels of malic acid, fumaric acid, lactic acid, gluconic acid, creatine, and hippuric acid were lower, while the levels of taurine, uric acid, and allantoin were higher in the hepatic tissues of feed-restricted ewes than those of ewes in the CON group.

### 2.4. Pathway Analysis of Differential Hepatic Metabolites

**Figure 4:**
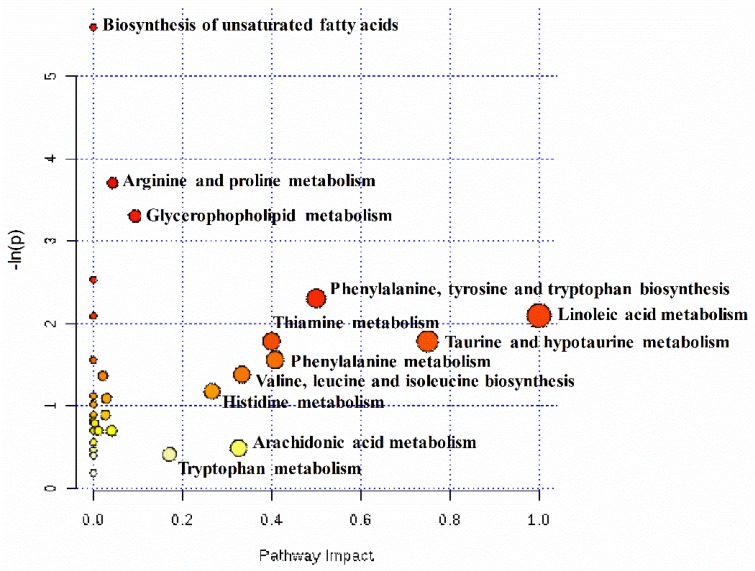
Metabolome view map of the differential metabolites identified in the hepatic tissues between the control group (CON, fed at the normal level, *n* = 8) and treated group (TR, restricted to a 30% level of feed intake, *n* = 8). Power calculation had identified a required sample size of 8 ewes per group in order to enable detection of an effect size of 1.94 for most of the cognitive test scores with 95% power and a type I error of 5%. The x-axis represents the pathway impact, and the y-axis represents the pathway enrichment. The larger size indicates higher pathway enrichment, and the darker color indicates higher pathway impact values.

To comprehensively evaluate how multiple pathways were altered when pregnant ewes were undernourished, an enrichment analysis was performed (Figure 4). The results demonstrated that unsaturated fatty acids biosynthesis, arginine and proline metabolism, and glycerophospholipid metabolism were enriched significantly (*p* < 0.05). In addition, the results of the pathway topology analysis demonstrated that nine pathways’ impact values, including linoleic acid metabolism (1.00), taurine and hypotaurine metabolism (0.75), phenylalanine, tyrosine and tryptophan biosynthesis (0.50), phenylalanine metabolism (0.41), thiamine metabolism (0.40), valine, leucine, and isoleucine biosynthesis (0.33), arachidonic acid metabolism (0.33), histidine metabolism (0.27), and tryptophan metabolism (0.17), were higher than the cutoff value of relevance (0.1).

As shown in Supplementary Figure S2, of the top ten enriched metabolic processes of differential metabolites identified in the hepatic tissues of the two groups, five were related to lipid metabolism, including the oxidation of very long-, long-, and branch-chain fatty acids, alpha linolenic acid and linoleic acid metabolism, and phospholipid biosynthesis. Two were linked to amino acid metabolism, including phenylalanine, tyrosine, and methylhistidine metabolism.

### 2.5. Correlation Networks of Hepatic Metabolites

**Figure 5:**
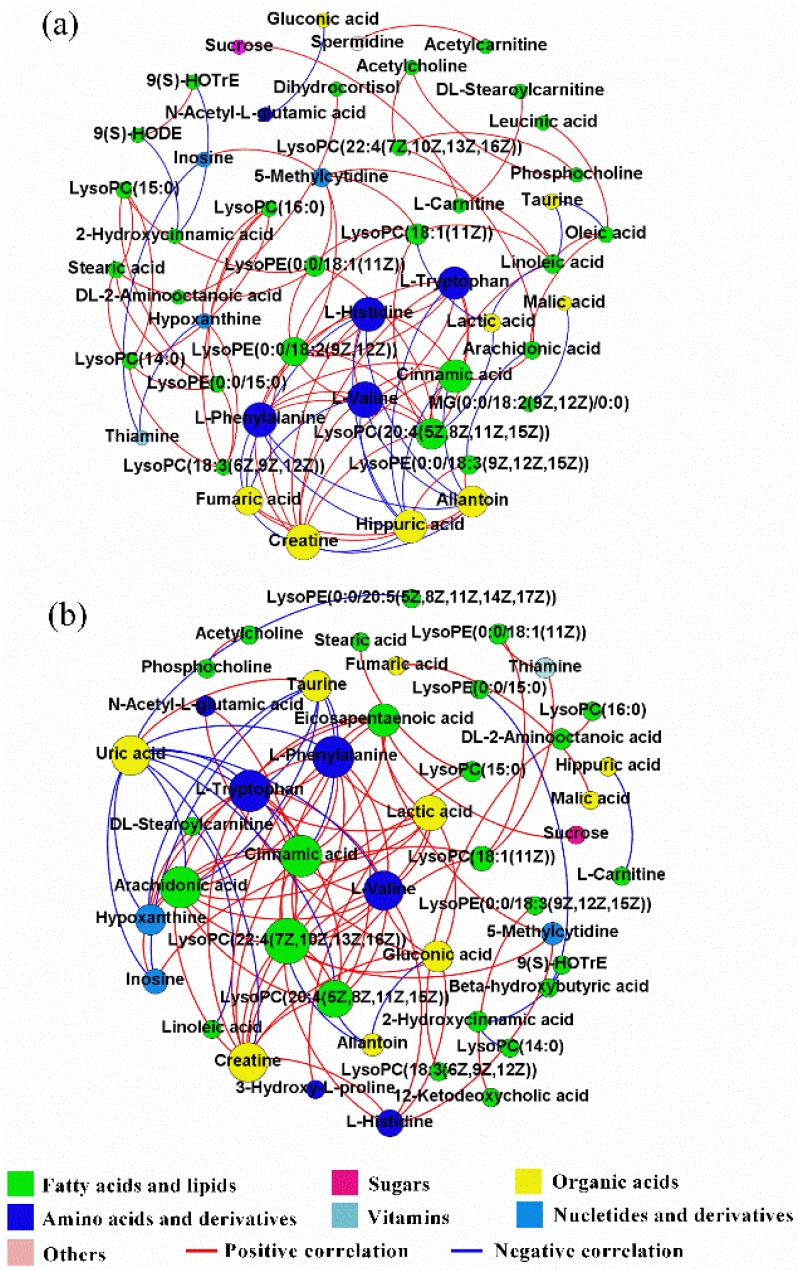
Correlation networks of hepatic metabolites for ewes in the control group (CON, fed at the normal level, *n* = 8) (**a**) and treated group (TR, restricted to a 30% level of feed intake, *n* = 8) (**b**) based on Spearman’s correlation coefficients (|r| > 0.8 and *p* < 0.05). Node size and color corresponds to the degree and classification, respectively. Red lines denote positive correlations, while blue lines denote negative correlations.

Correlation networks were utilized to express the comprehensive relationships between hepatic metabolites and to compare the overall metabolic profiles of the hepatic tissues of feed-restricted ewes (Figure 5b) and ewes in the CON group (Figure 5a). In the correlation networks, nodes indicate metabolites and edges indicate the correlations between metabolites. To obtain a deeper understanding of these two correlation networks, the connectivity and centrality measurement parameters, including eigenvector centrality, degree, and graph density, were calculated. Eigenvector centrality is the principal eigenvalue of the adjacency matrix of a correlation network, which reflects the vital degree of a node in the network. Degree refers to the number of edges incident upon this node, which is used to manifest the critical degree of each node. Graph density denotes the closeness of node connections.

The correlation network of differential metabolites for ewes in the CON group was comprised of 47 nodes and 97 edges, while that of feed-restricted ewes was consisted of 45 nodes and 97 edges. However, there were only 25 common edges between these two networks. Relative to ewes in the CON group, both the values of the average degree (4.128 VS. 4.311) and the graph density (0.090 VS. 0.098) of the correlation network for feed-restricted ewes were increased. The correlations of hepatic metabolites of ewes in the CON group were mainly centralized in amino acids and organic acids. Both l-valine and creatine had the highest eigenvector centrality (1.000), closely followed by l-phenylalanine (0.927), l-histidine (0.914), cinnamic acid (0.876), hippuric acid (0.866), and l-tryptophan (0.847). l-valine, creatine, and LysoPC(20:4(5*Z*,8*Z*,11*Z*,15*Z*)) had the highest degree (10), closely followed by l-histidine (9), l-phenylalanine (9), hippuric acid (9), and cinnamic acid (9). However, the correlations of hepatic metabolites in the feed-restricted ewes were mainly centralized in fatty acids and lipids and amino acids. Arachidonic acid, cinnamic acid, l-tryptophan, and l-phenylalanine had the highest eigenvector centrality (1.000), followed by LysoPC(22:4(7*Z*,10*Z*,13*Z*,16*Z*)) (0.948), l-valine (0.914), and creatine (0.914). LysoPC(22:4(7*Z*,10*Z*,13*Z*,16*Z*)) had the highest degree (14), followed by arachidonic acid (12), l-tryptophan (12), l-phenylalanine (12), cinnamic acid (12), l-valine (11), and creatine (11).

### 2.6. Expression of Genes Involved in Long-, Medium-, and Short-Chain Fatty Acids Metabolism

**Figure 6:**
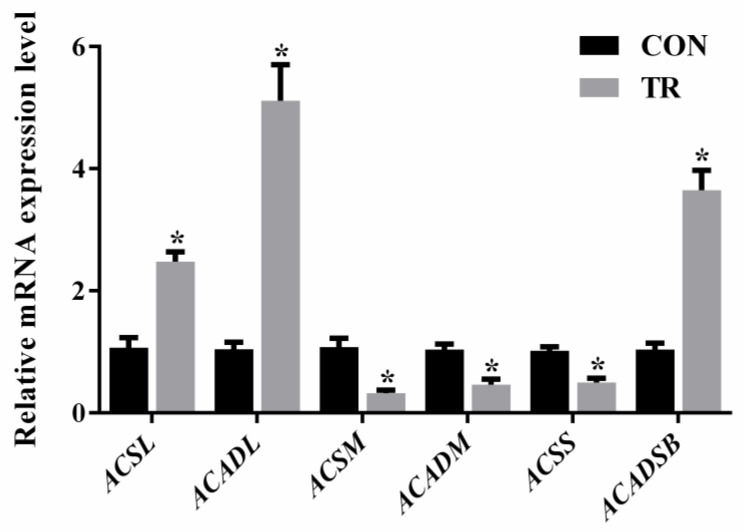
The gene expressions of acyl-CoA synthases and dehydrogenases in the hepatic tissues of ewes in the control group (CON, fed at the normal level, *n* = 10) and treated group (TR, restricted to a 30% level of feed intake, *n* = 10). Values are means with their standard errors represented by vertical bars. * Mean values with asterisks were significantly different between the two groups (*p* < 0.05, independent-sample *t*-test). *ACSL*, long-chain acyl-CoA synthase; *ACADL*, long-chain acyl-CoA dehydrogenase; *ACSM*, medium-chain acyl-CoA synthase; *ACADM*, medium-chain acyl-CoA dehydrogenase; *ACSS*, short-chain acyl-CoA synthase; *ACADSB*, short and branch-chain acyl-CoA dehydrogenase.

As shown in Figure 6, when compared with the CON group, the expression levels of long-chain acyl-CoA synthase (*ACSL*) and long-chain acyl-CoA dehydrogenase (*ACADL*) were increased, while that of medium-chain acyl-CoA synthase (*ACSM*) and medium-chain acyl-CoA dehydrogenase (*ACADM*) were decreased in the hepatic tissues of feed-restricted ewes. In addition, short-chain acyl-CoA synthase (*ACSS*) expression was lower, while short and branch-chain acyl-CoA dehydrogenase (*ACADSB*) was higher in the hepatic tissues of feed-restricted ewes than those of ewes in the CON group, respectively.

## 3. Discussion

As energy requirements for fetal growth and development exceed the dietary energy supply during late gestation, pregnant ewes are capable to change physiological metabolism and the synthesis of nutrients to adapt to the undernutrition [9]. However, enhanced body fat mobilization used to maintain energy homeostasis leads to the excessive genesis of ketones in the liver, which induce pregnancy toxemia [5,10]. Some previous studies restricted the feed intake of pregnancy ewes to 70% or 50% level to study the body’s response, but not all the ewes demonstrated pregnancy toxemia [11,12]. In the present study, 70% level of feed restriction was utilized to make it close to the spontaneous undernutrition and to develop undernutrition-induced pregnancy toxemia model. Our data showed that the concentration of plasma glucose was reduced while the concentrations of blood NEFAs and BHBA were excessively increased, and a series of clinical symptoms were observed in feed-restricted ewes, which confirmed the successful development of pregnancy toxemia in the current study [1]. In addition, as compared with the traditional strategies in feed restriction induced pregnancy toxemia experiment, the present study may provide more insights on the complex adaptive mechanisms of pregnant ewes to the undernutrition during late gestation.

### 3.1. Mobilization of Body Fat to Provide Energy

In the present study, both the PCA and the PLS-DA of hepatic metabolites revealed a clear separation between ewes in the CON and TR groups, implying a significant alternation in the metabolic profile of hepatic tissues when pregnant ewes were undernourished. In addition, 54 differential hepatic metabolites were identified between two groups, further demonstrated that severe feed restriction resulted in a significant alternation in hepatic metabolism. Enrichment analysis of differential metabolites revealed that, among the top ten enriched pathways, four were correlated with fatty acids oxidation, which suggested that severe feed restriction altered the hepatic fatty acid oxidation in ewes. Actually, the significant accumulation of BHBA, which is the end-product of incomplete oxidation of NEFAs, in the livers and blood of feed-restricted ewes further implied the intensified fatty acid oxidation. These findings are also similar to the results reported in mice with calorie restriction, which exhibit enhanced fatty acid oxidation compared with ad libitum-feed controls [13,14,15].

Interestingly, the present study showed that, among the differential NEFAs, the levels of all medium-chain fatty acids were reduced, while almost the levels of all long-chain fatty acids were increased in the hepatic tissues of feed-restricted ewes when compared with the controls. This could occur because it is much easier for medium-chain fatty acids to enter the mitochondria and participate in beta-oxidation, while long-chain fatty acids should be transported with the assistance of carnitine [16]. Consistently, l-carnitine, which plays an important role in importing acyl-CoA into the mitochondria, was markedly increased in the hepatic tissues of feed-restricted ewes. Meanwhile, the dramatically increased stearoylcarnitine levels indicated that a large amount of stearoyl-CoA combined with carnitine and entered the mitochondria for beta-oxidation. From the perspective of nurtigenomics, nutrients, as dietary signals, can be investigated by cellular sensor systems, thereby influence the expression of genes [17]. In the present study, our data showed that, along with the increase of long-chain fatty acids and the decrease of medium-chain fatty acids, *ACSL* and *ACADL* expressions were up-regulated while *ACSM* and *ACADM* expressions were down-regulated, implying the enhanced oxidation of long-chain fatty acids and the reduced oxidation of medium-chain fatty acids in the hepatic tissues of feed-restricted ewes. For short and branch-chain fatty acids, the up-regulated *ACADSB* expression and the down-regulated *ACSS* expression might suggest the intensified oxidation of branch-chain fatty acids in the livers of feed-restricted ewes, which was consistent with the enrichment analysis of differential metabolites. Previous studies show that acetylcarnitine plays an important buffering role in transporting acetyl-CoA from within the mitochondria to outside when the amount of acetyl-CoA exceeds the utilization of the tricarboxylic acid cycle (TCA) [18,19,20]. In the present study, the level of acetylcarnitine in the hepatic tissues of feed-restricted ewes was 135.3-fold higher than that of the CON group, indicating that a large amount of acetyl-CoA might be accumulated in the mitochondria. In addition, NEFAs can also be esterified to triglycerides with glycerol. In the present study, largely amounts of fat vacuoles in hepatic histological sections suggested that the levels of NEFAs overwhelmed the metabolic capacity of livers in feed-restricted ewes. Excessively accumulated triglycerides might induce hepatic steatosis and cause the appearance of deep yellow and swollen. Furthermore, HODE and HOTrE are not only long-chain fatty acids, they are also primary lipid oxidation products that can produce secondary lipid oxidation products. In the body, these observations suggested potential oxidative stress. Additionally, MG is produced through the release of a fatty acid from diacylglycerol by hormone sensitive lipase or diacylglycerol lipase. The marked elevation of MG (0:0/18:2(9*Z*,12*Z*)/0:0) might suggest the mobilization of hepatic triglycerides in feed-restricted ewes. Thus, our results showed that both the processes of triglyceride synthesis and degradation were disrupted in the livers of feed-restricted ewes.

Another compelling finding in the current study was that a great number of LysoPCs and LysoPEs were accumulated in the livers of feed-restricted ewes. LysoPCs and LysoPEs are the degraded products of phosphatidylcholines and phosphatidylethanolamines, respectively, so the excessive increasing of them might indicate the enhanced mobilization of hepatic phospholipids when pregnant ewes were undernourished. As the phosphatidylcholines and phosphatidylethanolamines are major membrane lipids [21,22], the disordered phospholipid metabolism might cause the alternation of membrane’s structure and function. 

### 3.2. Utilization of Free Amino Acids to Provide Energy

**Figure 7:**
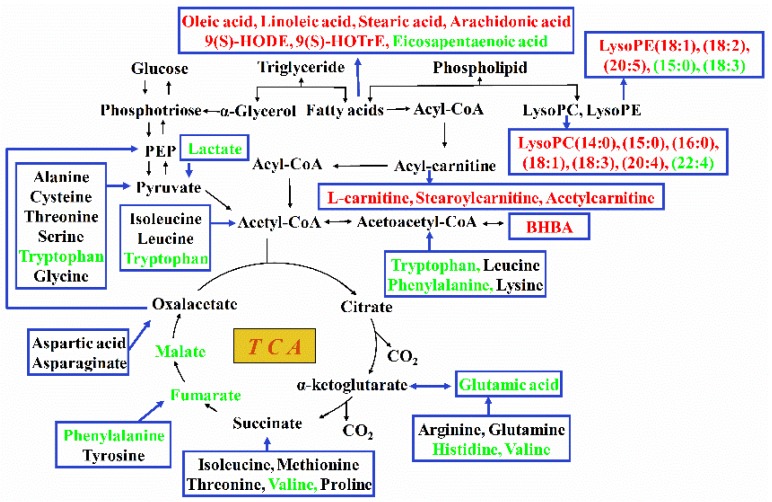
Overview of metabolic alteration related to energy conversion in the hepatic tissues of feed-restricted ewes during late gestation. The red font indicates the increased metabolites while the green font indicates the decreased metabolites in feed-restricted ewes compared with ewes in the control group. TCA, tricarboxylic acid cycle; LysoPC, lysophosphatidyl choline; LysoPE, lysophosphatidyl ethanolamine; BHBA, beta-hydroxybutyric acid; PEP, phosphoenolpyruvate.

Apart from body fat mobilization, body protein can also be mobilized to relieve a continuous negative energy balance [23]. Interestingly, for the seven differential amino acids and derivatives examined, six of them decreased in the hepatic tissues of feed-restricted ewes. The only increased amino acid derivative (3-hydroxy-l-proline) in the hepatic tissues of feed-restricted ewes was the metabolic by-product of l-proline. During this reaction, under the catalysis of proline 3-hydroxylase, l-proline, oxygen, and 2-oxoglutarate are converted to *cis*-3-hydroxy-l-proline, carbon dioxide, and succinate [24]. Similarly, some amino acids can be converted to the intermediate substances of TCA, gluconeogenesis, and ketogenesis (Figure 7). In the present study, the increased level of 3-hydroxy-l-proline and the decreased levels of l-histidine, l-valine, *N*-acetyl-l-glutamic acid, l-tryptophan, and l-phenylalanine indicated the likelihood that some amino acids in the hepatic tissues of feed-restricted ewes had been metabolized to provide energy for body. Moreover, the fetus-placental energy requirement might represent 72% of the supply of maternal amino acids [25], which could be another important reason for the dramatic decrease in the levels of amino acids in maternal body. Furthermore, the significant reduction in the levels of sucrose, fumaric acid, malic acid, lactic acid, and gluconic acid indicated that energy substrates were still extremely short in feed-restricted ewes though the utilization of fatty acids and amino acids.

### 3.3. Metabolic Dysfunction of Nucleotides and Derivatives, Vitamins, and Organic Acids

The decrease in the levels of hypoxanthine, 5-methylcytidine, inosine, and thiamine implied metabolic dysfunction of nucleotides and derivatives and vitamins in the hepatic tissues of feed-restricted ewes. Uric acid is mainly formed from xanthine and hypoxanthine under the catalysis of xanthine oxidase. Subsequently, uricase further oxidizes uric acid to produce allantoin [26]. A high level of uric acid and allantoin is associated with metabolic diseases including gout, diabetes, and kidney stones. In the present study, high levels of uric acid and allantoin in the hepatic tissues of feed-restricted ewes might suggest the metabolic disruption of purines. Chenodeoxycholic acid, which is a main bile acid, is synthesized from cholesterol in the liver [27]. Taurine is a major component of bile, which is conjugated via its amino terminal group with cholic acid and chenodeoxycholic acid and produces the two species of bile salts, taurocholate and taurochenodeoxycholate, respectively [28]. In addition, 12-ketodeoxycholic acid is a kind of bile acid. The alteration in the levels of chenodeoxycholic acid, taurine, and 12-ketodeoxycholic acid found in the current study might imply the disrupted bile acid metabolism and bile genesis in the hepatic tissues of feed-restricted ewes. A previous study also showed that short-term calorie restriction increased bile acids content in the livers of male mice possibly via enhanced bile acid synthesis and conjugation in liver as well as intracellular transport in ileum [29].

### 3.4. The Change in the General Metabolic Pattern

It should be noted that the metabolic profile has proven that feed restriction during late gestation alters the levels of some hepatic metabolites when compared with ewes in the CON group. However, a given hepatic metabolite doesn’t exist independently, its alteration may be relevant to the changes of other metabolites, and the correlation network of hepatic metabolites can demonstrate the overall metabolic process that occurs in the liver. The greater values of network density and average degree in feed-restricted ewes indicated that feed restriction during late gestation enhanced the hepatic metabolic connection. Combined with the detailed metabolites and correlation networks, the above results demonstrated that feed restriction during late gestation possibly disrupted the originally metabolic balance, which was principally centralized in amino acids and organic acids. Furthermore, a new adaptive mechanism that is primarily centralized in fatty acids and lipids and amino acids was developed. The correlation networks of hepatic metabolites offer novel knowledge to obtain a comprehensive understanding of the complex metabolism in the liver.

## 4. Materials and Methods

### 4.1. Animal and Experimental Design

The animal experimental design and procedures were approved by the Animal Care and Use Committee of Nanjing Agricultural University (SYXK(Su)2015-0656). This experiment was performed as part of a lager project designed to study the metabolic changes of carbohydrates, lipids, and proteins in maternal and fetal livers of pregnant sheep. The animal management was previously described by Xue et al. [30]. Briefly, twenty pregnant Hu-sheep with similar parity and body status, who were pregnant for 108 days and carrying two or more fetuses, were enrolled in the current study. After 7 days of preparatory feeding (ad libitum access to total mixed ration), they were assigned to two groups randomly for a 15-day intervention. Ewes in the CON group (*n* = 10) were fed normally (metabolic energy 18.16 MJ/d) according to the feed intake calculated during the adaptive period, while ewes in the TR group (*n* = 10) were restricted to the 30% level (metabolic energy 5.45 MJ/d). All ewes were fed two times a day (9:00 & 15:00) in individual pens and had free access to water. Ingredients and compositions of the total mixed ration used in this animal experiment are shown in Supplementary Table S1.

### 4.2. Collection of Blood and Hepatic Tissue Samples

Blood samples were collected from the jugular vein prior to morning feeding before and after the experiment, to investigate the levels of glucose, NEFAs, and BHBA. After 15-day intervention, all ewes were sacrificed through bloodletting slaughter at 4 h after morning feeding. Subsequently, internal organs including liver, heart, spleen, kidney, and uterus were taken out and weighed respectively to calculate the organ indexes (e.g., liver index is the proportion of liver weight in ewe’s live weight). Hepatic tissue samples were collected from the same location in each liver within 30 min. Samples were immediately frozen in liquid nitrogen for the LC/MS analysis and quantitative real-time polymerase chain reaction (PCR). Meanwhile, another part of hepatic tissue for each ewe was collected and rapidly fixed in 4% paraformaldehyde to make hematoxylin-eosin staining section.

### 4.3. Analysis of Biochemical Indicators in Blood

The Free-style Optium Blood Glucose and Ketone Monitoring System (Abbott Diabetes Care Ltd., Oxon, UK) was used to determine the BHBA level in blood. The glucose and NEFAs levels in the blood were measured by available kits (Jian-Cheng Bioengineering Institute, Nanjing, China) following the manufacturer’s instructions. 

### 4.4. Histological Analysis of the Livers

Histological sections of hepatic tissues were made according to the methodology described by Odongo et al. [31]. In short, hepatic tissues were fixed in 4% paraformaldehyde (Sigma, St. louis, MO, USA), following embedded in paraffin to make specimens. Subsequently, specimens were sectioned at 6-μm thickness and stained with hematoxylin-eosin, lastly mounted for analysis under light microscope.

### 4.5. Sample Preparation for LC/MS Detection

Eight ewes’ hepatic samples in each group were randomly selected to carry out metabolome analysis. Hepatic tissue extracts used in LC/MS-based metabolomics were prepared using the following method. Briefly, 50 mg of the hepatic tissue sample, 300 μL methanol (Merck, Dannstadt, Germany), and 10 μL l-2-chlorophenylalanine (interior label) were added to a 1.5 mL eppendorf tube, which was mixed on Votex-5 (Kylin-Bell Lab Instruments Co., LTD., Haimen, China) for 30 s, homogenized by Homogenizer Scientz-48 (Scientz Biotechnology Co., LTD., Ningbo, China) for 2 min, then centrifuged for 10 min at 4 °C, 13, 800 *g*. After that, 200 μL of the supernatant was transferred to a new LC/MS glass vial.

### 4.6. LC/MS-Based Hepatic Metabolic Profiling

The Exactive Orbitrap MS (Thermo Fisher Scientific, Bremen, Germany) interfaced with a heated electrospray ionization source, and the orbitrap mass analyzer was operated at 35,000 mass resolution. The separation conditions of the chromatographic column (Hypergod C18 (100 mm × 4.6 mm 3 μm)) were as follows: Column temperature was 40 °C, and flow rate was 0.3 mL/min. Mobile phase A was water and 0.1% formic acid (CNW, Duesseldorf, Germany) while mobile phase B was acetonitrile (Merck, Dannstadt, Germany) and 0.1% formic acid. The gradient of mobile phase was shown in Supplementary Table S2. The volume of the injected sample was 4 μL, and autosampler temperature was 4 °C. The parameters of the MS were as follows: Heater temperature 300 °C, sheath gas flow rate 45 arb, aux gas flow rate 15 arb, sweep gas flow rate 1 arb, spray voltage 3.0 KV, capillary temperature 350 °C, and S-Lens RF level 30%. The Kyoto Encyclopedia of Genes and Genomes Database (http://www.genome.jp/kegg) and the online Human Metabolome Database (http://www.hmdb.ca) were utilized to align the molecular mass data to identify metabolites. The metabolite name was reported if the difference between theoretical mass and observed mass was less than 10 ppm. Then, the method of isotopic distribution measurement was used to further validate these matched metabolites. Commercial reference standards were utilized to validate and confirm the hepatic metabolites with high confidence by comparison of their MS/MS spectra and retention time.

### 4.7. RNA Extraction, Purification, and Complementary DNA Synthesis

Total RNA was isolated from frozen fetal liver samples using the TRIzol method (Takara Bio, Otsu, Japan) following the instructions of Chomczynski and Sacchi [32]. The quality of the RNA extracted was also verified by a Nanodrop spectrophotometer ND-1000UV-Vis (Thermo Fisher Scientific, Madison, WI, USA) to ensure all RNA samples had an absorbance (A260/A280, A260/A230) between 1.8 and 2.0. All RNA solution samples were diluted to the same concentration (500 ng/µL). Total RNA (1 µg) was reversely transcribed to complementary DNA by a PrimeScript reverse transcription kit (Takara Bio, Otsu, Japan) using the two-step method (with genomic DNA eraser).

### 4.8. Quantitative Real-Time PCR

Quantitative PCR was carried out to analyze the mRNA expression levels of genes linked to lipid metabolism in fetal livers. We used Primer 5 software (Whitehead Institute, Cambridge, MA, USA) and the BLAST computer program (National Center for Biotechnology Information, Bethesda, MD, USA) to design the primers. Details of the primer sets used for the PCR are listed in Supplementary Table S3. The PCR premix was a 20 µL reaction system, containing 10 µL SYBRR Premix Ex TaqTM (TLi RNase H Plus) (2*), 0.4 µL forward primer (10 µM), 0.4 µL reverse primer (10 µM), 0.4 µL ROX Reference Dye (50*), 2.0 µL complementary DNA template, and 6.8 µL ddH2O. The thermocycling conditions were as follows: 95 °C for 30 s for denaturation and the activation of Taq polymerase, followed by 40 thermal cycles of 95 °C for 5 s and 60 °C for 34 s. After the amplification process, a melting curve analysis was performed by heating the plate (60–99 °C with a heating rate of 0.1 °C/s and a fluorescence measurement). Fluorescence was detected by the QuantStudio 5 Real-Time PCR Instrument (Thermo Fisher Scientific, Waltham, MA, USA). The mRNA level of each studied gene was analyzed according to the 2^−ΔΔCt^ method (normalized to β-actin).

### 4.9. Data Analysis

Significant tests on blood indicators were performed using the independent-samples *t*-test in SPSS 19.0. The data of LC-MS were analyzed on SIEVE software (Thermo Fisher Scientific, Waltham, MA, USA), then were normalized and edited into two-dimensional data matrix in Excel 2016. The PCA and PLS-DA were performed using SIMCA-P 13.0 software (Umetrics, Umea, Sweden). The FC was the ratio of the level of corresponding metabolite obtained from the two groups (TR/CON). Differential metabolites were selected by combing VIP generated in PLS-DA as well as FDR (corrected *p* value) and FC obtained in statistical analysis (VIP > 1, FDR < 0.05, and FC > 1.5 or < 0.67). Then, the data of differential metabolites were submitted to the MetaboAnalyst web server (http://www.metaboanalyst.ca) to examine metabolic pathway distribution and to conduct an enrichment analysis [33]. The Spearman’s correlation coefficients (r) among the metabolites were calculated with SPSS 19.0 and a significance threshold of *p* < 0.05 and |r| > 0.8 was adopted. Gephi 0.8.2 software [34] was used to visualize the correlation network of metabolites.

## 5. Conclusions

Severe feed restriction caused serious lipid metabolism disorder and even altered the general metabolic correlations in livers of pregnant ewes. Fatty acids, especially long-chain fatty acids, were accumulated in livers of feed-restricted ewes and were oxidized to provide energy. Some amino acids and organic acids might also be utilized to relieve the energy shortage in feed-restricted ewes.

## Figures and Tables

**Table 1:** Differential metabolites identified in the hepatic tissues of ewes between the control group (CON, fed at the normal level, *n* = 8) and treated group (TR, restricted to a 30% level of feed intake, *n* = 8). VIP, variable importance in projection; FDR, false discovery rate; FC, fold change (TR/CON); BHBA, beta-hydroxybutyric acid; LysoPC, lysophosphatidyl choline; LysoPE, lysophosphatidyl ethanolamine; MG, monoacylglycerol; 9(*S*)-HODE, 9*S*-hydroxy-10*E*, 12*Z*-octadecadienoic acid; 9(*S*)-HOTrE, 9*S*-hydroxy-10*E*,12*Z*,15*Z*-octadecatrienoic acid.

Name	Mass	VIP	FDR	FC
**Fatty acids and lipids**	-	-	-	-
BHBA	104.04213	1.126	0.033	1.593
dl-2-Aminooctanoic acid	159.12537	1.364	0.004	0.534
Cinnamic acid	148.05241	1.162	0.018	0.608
2-Hydroxycinnamic acid	163.06326	1.337	0.005	0.591
Eicosapentaenoic acid	302.22404	1.346	0.005	0.482
9(*S*)-HODE	296.23475	1.685	<0.001	6.530
9(*S*)-HOTrE	294.21894	1.569	0.002	10.016
Oleic acid	282.25560	1.661	<0.001	3.807
Linoleic acid	280.23921	1.661	<0.001	3.418
Stearic acid	284.27116	1.539	<0.001	1.778
Arachidonic acid	304.23997	1.434	0.002	2.589
Acetylcarnitine	203.11522	1.192	0.027	135.264
l-Carnitine	161.10419	1.589	0.001	7.205
dl-Stearoylcarnitine	427.36354	1.571	0.002	14.803
Acetylcholine	145.10966	1.300	0.013	1.997
Phosphocholine	183.06569	1.301	0.013	2.002
LysoPC(14:0)	467.29894	1.141	0.021	1.629
LysoPC(15:0)	481.31638	1.540	<0.001	1.611
LysoPC(16:0)	495.32976	1.287	0.007	1.685
LysoPC(18:1(11*Z*))	521.34465	1.078	0.028	1.750
LysoPC(18:3(6*Z*,9*Z*,12*Z*))	517.31108	1.564	<0.001	2.017
LysoPC(20:4(5*Z*,8*Z*,11*Z*,15*Z*))	544.33000	1.383	0.003	2.043
LysoPC(22:4(7*Z*,10*Z*,13*Z*,16*Z*))	571.36009	1.071	0.035	0.484
LysoPE(0:0/15:0)	439.26760	1.184	0.025	0.261
LysoPE(0:0/18:1(11*Z*))	479.29875	1.322	0.006	2.119
LysoPE(0:0/18:2(9*Z*,12*Z*))	477.28301	1.121	0.024	1.527
LysoPE(0:0/18:3(9*Z*,12*Z*,15*Z*))	474.24544	1.190	0.015	0.503
LysoPE(0:0/20:5(5*Z*,8*Z*,11*Z*,14*Z*,17*Z*))	499.26766	1.250	0.015	1.736
MG(0:0/18:2(9*Z*,12*Z*)/0:0)	354.27562	1.211	0.013	3.078
Chenodeoxycholic acid	392.29152	1.375	0.006	1.983
12-Ketodeoxycholic acid	390.23747	1.087	0.027	0.304
Dihydrocortisol	364.21602	1.068	0.030	0.576
Leucinic acid	132.07866	1.213	0.024	0.598
**Amino acids and derivatives**	-	-	-	-
l-Histidine	155.06909	1.429	0.002	0.540
l-Valine	117.07830	1.105	0.025	0.561
l-Tryptophan	204.08893	1.226	0.013	0.575
l-Phenylalanine	165.07807	1.146	0.020	0.639
*N*-Acetyl-l-glutamic acid	189.05966	1.269	0.008	0.330
3-Hydroxy-l-proline	131.05827	1.441	0.007	4.862
**Sugars**	-	-	-	-
Sucrose	342.11511	1.134	0.028	0.120
**Organic acids**	-	-	-	-
Malic acid	134.02162	1.392	0.006	0.567
Fumaric acid	116.01103	1.165	0.018	0.594
Lactic acid	90.03218	1.574	<0.001	0.570
Gluconic acid	196.05829	1.464	0.001	0.366
Creatine	131.07292	1.047	0.033	0.470
Hippuric acid	179.05806	1.107	0.025	0.582
Taurine	125.01437	1.088	0.028	2.566
Uric acid	168.02820	1.356	0.004	1.777
Allantoin	158.04402	1.137	0.033	3.428
**Nucleosides and nucleotides**	-	-	-	-
Hypoxanthine	136.03803	1.405	0.007	0.413
5-Methylcytidine	257.10237	1.504	<0.001	0.426
Inosine	268.08027	1.259	0.011	0.432
**Vitamins**	-	-	-	-
Thiamine	264.06179	1.405	0.008	0.255
**Others**	-	-	-	-
Spermidine	145.15767	1.151	0.020	2.380

## Supplementary Materials

The following are available online at http://www.mdpi.com/2218-1989/8/4/85/s1. Table S1. Details of total mixed ration fed to ewes in animal experiment. Table S2. The gradient of mobile phase. Table S3. Gene primers used for quantitative real-time PCR. Figure S1. Hepatic histomorphological analysis of ewes in the control group (CON, fed at the normal level) and treated group (TR, restricted to a 30% level of feed intake). (a) Hepatic appearance of ewes in the CON and TR groups. Hematoxylin and eosin staining sections of hepatic tissues for ewes in the CON group (b) and TR group (c). Figure S2. Enrichment analysis of metabolic processes for the differential metabolites identified in the hepatic tissues between the control group (CON, fed at the normal level, *n* = 8) and treated group (TR, restricted to a 30% level of feed intake, *n* = 8).

## References

[1] Cal-Pereyra L., Acosta-Dibarrat J., Benech A., Silva S.D., Martín A., González-Montańa J.R. (2012). Ewe pregnancy toxemia. Rev. Mex. Cienc. Pecu..

[2] Andrews A. (1997). Pregnancy toxemia in ewe. Practice.

[3] Gao F., Hou X.Z., Liu Y.C. (2007). Effect of hormonal status and metabolic changes of restricted ewes during late pregnancy on their fetal growth and development. Sci. China.

[4] Gluckman P.D., Cutfield W., Hofman P., Hanson M.A. (2005). The fetal, neonatal, and infant environments-the long-term consequences for disease risk. Early Hum. Dev..

[5] Sargison N.D., Scott P.R., Penny C.D., Pirie R.S., Kelly J.M. (1994). Plasma enzymes and metabolites as potential prognostic indices of ovine pregnancy toxaemia—A preliminary study. Br. Vet. J..

[6] Rook J.S., Herdt T.H. (2000). Pregnancy toxemia of ewes, does, and beef cows. Vet. Clin. N. Am. Food Anim. Pr..

[7] Van Saun R.J. (2000). Pregnancy toxemia in a flock of sheep. J. Am. Vet. Med. Assoc..

[8] Lima M.S., Pascoal R.A., Stilwell G.T. (2012). Glycaemia as a sign of the viability of the foetuses in the last days of gestation in dairy goats with pregnancy toxaemia. Ir. Vet. J..

[9] Reynolds L.P., Caton J.S., Redmer D.A., Grazul-Bilska A.T., Vonnahme K.A., Borowicz P.P., Luther J.S., Wallace J.M., Wu G., Spencer T.E. (2006). Evidence for altered placental blood flow and vascularity in compromised pregnancies. J. Physiol..

[10] Pethick D.W., Lindsay D.B. (1982). Metabolism of ketone bodies in pregnant sheep. Br. J. Nutr..

[11] Lacetera N., Bernabucci U., Ronchi B., Nardone A. (2001). Effects of subclinical pregnancy toxemia on immune responses in sheep. Am. J. Vet. Res..

[12] Cal-Pereyra L., Benech A., González-Montaña J., Acosta-Dibarrat J., Da-Silva S., Martín A. (2015). Changes in the metabolic profile of pregnant ewes to an acute feed restriction in late gestation. N. Z. Vet. J..

[13] Cao S.X., Dhahbi J.M., Mote P.L., Spindler S.R. (2001). Genomic profiling of short- and long-term caloric restriction effects in the liver of aging mice. Proc. Natl. Acad. Sci. USA.

[14] Chen D., Bruno J., Easlon E., Lin S.J., Cheng H.L., Alt F.W., Guarente L. (2008). Tissue-specific regulation of SIRT1 by calorie restriction. Genes Dev..

[15] Tsuchiya T., Dhahbi J.M., Cui X., Mote P.L., Bartke A., Spindler S.R. (2004). Additive regulation of hepatic gene expression by dwarfism and caloric restriction. Physiol. Genom..

[16] Dann H.M., Drackley J.K. (2005). Carnitine palmitoyltransferase I in liver of periparturient dairy cows: Effects of prepartum intake, postpartum induction of ketosis, and periparturient disorders. J. Dairy Sci..

[17] Müller M., Kersten S. (2003). Nutrigenomics: Goals and strategies. Nat. Rev. Genet..

[18] Lopaschuk G.D., Gamble J. (1994). Acetyl-CoA carboxylase: An important regulator of fatty acid oxidation in the heart. Can. J. Physiol. Pharmacol..

[19] Kiens B. (2006). Skeletal muscle lipid metabolism in exercise and insulin resistance. Physiol. Rev..

[20] Stephens F.B., Constantin-Teodosiu D., Greenhaff P.L. (2007). New insights concerning the role of carnitine in the regulation of fuel metabolism in skeletal muscle. J. Physiol..

[21] Carman G.M., Vance D.E., Vance J. (2008). Biochemistry of Lipids, Lipoproteins, and Membranes.

[22] Wellner N., Diep T.A., Janfelt C., Hansen H.S. (2013). N-acylation of phosphatidylethanolamine and its biological functions in mammals. Biochim. Biophys. Acta.

[23] Kokkonen T., Taponen J., Anttila T., Syrjäläqvist L., Delavaud C., Chilliard Y., Tuori M., Tesfa A.T. (2005). Effect of body fatness and glucogenic supplement on lipid and protein mobilization and plasma leptin in dairy cows. J. Dairy Sci..

[24] Mori H., Shibasaki T., Yano K., Ozaki A. (1997). Purification and cloning of a proline 3-hydroxylase, a novel enzyme which hydroxylates free l-proline to cis-3-hydroxy-l-proline. J. Bacteriol..

[25] Bell A.W. (1995). Regulation of organic nutrient metabolism during transition from late pregnancy to early lactation. J. Anim. Sci..

[26] Angstadt C.N. (1997). Purines and Pyrimidine Metabolism. Purine catabolism. NetBiochem.

[27] Russell D.W. (2003). The enzymes, regulation, and genetics of bile acid synthesis. Annu. Rev. Biochem..

[28] Shefer S., Hauser S., Lapar V., Mosbach E.H. (1973). Regulatory effects of sterols and bile acids on hepatic 3-hydroxy-3-methylglutaryl CoA reductase and cholesterol 7alpha-hydroxylase in the rat. J. Lipid Res..

[29] Fu Z.D., Klaassen C.D. (2013). Increased bile acids in enterohepatic circulation by short-term calorie restriction in male mice. Toxicol. Appl. Pharmacol..

[30] Xue Y.F., Guo C.Z., Hu F., Sun D.M., Liu J.H., Mao S.Y. (2018). Molecular mechanisms of lipid metabolism disorder in livers of ewes with pregnancy toxemia. Animal.

[31] Odongo N., AlZahal O., Lindinger M., Duffield T., Valdes E., Terrell S., McBride B. (2006). Effects of mild heat stress and grain challenge on acid-base balance and rumen tissue histology in lambs. J. Anim. Sci..

[32] Chomczynski P., Sacchi N. (1987). Single-step method of RNA isolation by acid guanidinium thiocyanate-phenol-chloroform extraction. Anal. Biochem..

[33] Xia J., Psychogios N., Young N., Wishart D.S. (2009). MetaboAnalyst: A web server for metabolomic data analysis and interpretation. Nucleic Acids Res..

[34] Bastian M., Heymann S., Jacomy M. Gephi: An open source software for exploring and manipulating networks. Proceedings of the Third International ICWSM Conference.

