# Supplementary material for: Hepatic Metabolic Profile Reveals the Adaptive Mechanisms of Ewes to Severe Undernutrition during Late Gestation
Source: Metabolites. 2018 Nov 27;8(4):85. doi: 10.3390/metabo8040085 (PMC6316483; doi:10.3390/metabo8040085)
Supplement: Supplementary file 1 [file metabolites-08-00085-s001.pdf]

SUPPLEMENTARY MATERIALS

Hepatic metabolic profile reveals the adaptive mechanisms of ewes to severe undernutrition during late gestation

Yanfeng Xue, Changzheng Guo, Fan Hu, Junhua Liu, Shengyong Mao*

Jiangsu Key Laboratory of Gastrointestinal Nutrition and Animal Health, Laboratory of Gastrointestinal Microbiology, College of Animal Science and Technology, Nanjing Agricultural University, Nanjing 210095, Jiangsu Province, China

Author E-mails:
Yanfeng Xue, xueyanfeng1990@163.com
Changzheng Guo, 2017205025@njau.edu.cn
Fan Hu, 2016105046@njau.edu.cn
Junhua Liu, liujunhua0011@163.com
* Correspondence: maoshengyong@njau.edu.cn; Tel.: (+00)86 25 8439 5523

Supplementary Table S1. Details of total mixed ration fed to ewes in animal experiment

Item	Diet
Ingredient composition, % DM[1]	
Rye silage	42.3
Oat hay	34.6
Maize	12.0
Soybean	5.8
Barley	4.2
Premix[2]	1.1
Nutrient composition	
Metabolic energy, MJ/kg DM	11.64
Crude protein, % DM	14.71
Crude fat, % DM	2.95
Neutral detergent fiber, % DM	48.32
Acid detergent fiber, % DM	29.09
Crude ash, % DM	7.35
Calcium, % DM	0.50
Phosphorus, % DM	0.37

[1] DM, dry matter basis.

[2] The premix (per kg) contained: Vitamin A 64.8 mg, Vitamin D_2 1.35 mg, Vitamin E 1080 mg, Nicotinic acid 353 mg, Cu 540 mg, Mn 537 mg, Zn 2422 mg, I 32 mg, Co 7.2 mg, Se 18 mg, Ca 130 g, P 66g, NaCl 140~180 g, $NaHCO_3$ 89 g.

Supplementary Table S2. The gradient of mobile phase

Time (min)	Flow rate (mL/min)	A (%)	B (%)
0	0.3	95	5
2	0.3	95	5
12	0.3	5	95
15	0.3	5	95
17	0.3	95	5

Supplementary Table S3. Gene primers used for quantitative real-time PCR

Gene symbol	Primer sequence	Accession number	Product size (bp)
ACSL	F: GAACTACAGGCAACCCCAAAG<br>R: TGCAAGCGGTAAAAACGAAA	XM_015104563.1	137
ACADL	F: TCAGTAATGGGTGGCTGTGTG<br>R: ATTCTGCAGTATCCTGGGCTT	XM_012156500.1	171
ACSM	F: AATGGAAGGTTCTGGCTGGA<br>R: GGGAAACTCGGAAAGTGTTTG	XM_004020818.3	183
ACADM	F: GGCGAGTACCCTGTTCCATT<br>R: CCTCAGTCATTCTCCCCAAAT	GAAI_01002329.1	244
ACSS	F: GGCTGGGGAACGATGTGAT<br>R: CCCTGGAGGAGGTGTTTTAGA	XM_015094557.1	151
ACADSB	F: CTGGGCTGTTCGTGGTGAT<br>R: AATGTGATTGGGCAGGTGG	XM_012103099.2	154
β-actin	F: TCGTGATGGACTCTGGGGA<br>R: GCCGTGGTGGTGAAGCTGTA	JN_033788.1	160

Supplementary Figure S1.

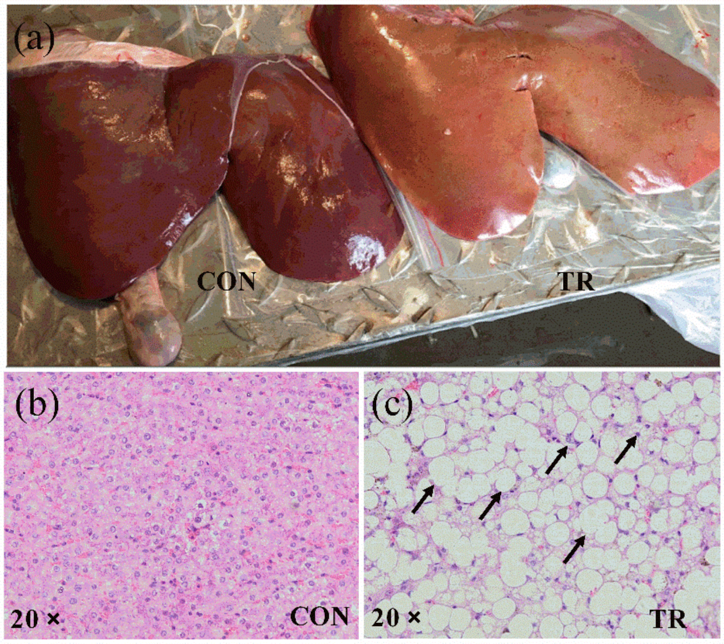

Supplementary Figure S1. Hepatic histomorphological analysis of ewes in the control group (CON, fed at the normal level) and treated group (TR, restricted to a 30% level of feed intake). (a)Hepatic appearance of ewes in the CON and TR groups. Hematoxylin and eosin staining sections of hepatic tissues for ewes in the CON group (b) and TR group (c).

Supplementary Figure S2.

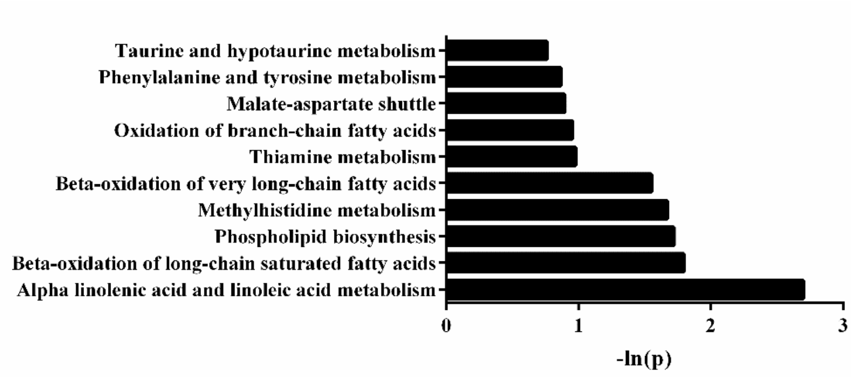

Supplementary Figure S2. Enrichment analysis of metabolic processes for the differential metabolites identified in the hepatic tissues between the control group (CON, fed at the normal level, n=8) and treated group (TR, restricted to a 30% level of feed intake, n=8).
